# THE INTERVIEW FOR DECISIONAL ABILITIES: DEVELOPING THE EVIDENCE BASE FOR ADULT PROTECTIVE SERVICES

**DOI:** 10.1093/geroni/igz038.1854

**Published:** 2019-11-08

**Authors:** Theresa Sivers-Teixeira, Gregory Stevens, Kelly Sadamitsu, Christina Penate, Bonnie Olsen

**Affiliations:** USC Keck School of Medicine, Los Angeles, California, United States

## Abstract

Adult Protective Services (APS) workers assess clients for abuse and neglect and are asked to determine the client’s understanding of risks they face. Yet, APS workers have little structured training in how to make such judgements. The Interview for Decisional Abilities (IDA 3.0-CA) is a tool designed for use by APS workers to assess the ability of suspected victims of elder mistreatment to make decisions about the risks they face. This study evaluates the impact of training and use of this tool on the knowledge, experiences and ability of APS workers to determine decisional ability. APS workers and supervisors were recruited from central and northern California APS programs and randomized into either control (n=94) or IDA 3.0-CA training groups (n= 95). Baseline surveys measure knowledge of, and experiences with, assessing decisional ability and determining next steps for case management. Additionally, respondents determine the decisional ability of three case scenarios. Three months post-training, controls and trained subjects complete the same survey with a new set of cases. Preliminary results at baseline indicate there were no statistically-significant differences between trained subjects (n=42) and controls (n=50) in their knowledge scores (78.6% correct vs. 81.0%, p=0.6641) or performance assessing decisional ability in the case scenarios (60.1% correct vs. 63.3%, p=0.3497). Reported experiences assessing decisional ability and determining next steps in case management were also similar for trained subjects and controls. Complete results will be presented regarding change in knowledge scores, experiences, and assessing decisional ability in case scenarios compared across trained subjects and controls.

